# *Greek Fire, Poison Arrows, and Scorpion Bombs*: How Tumor Cells Defend Against the Siege Weapons of Cytotoxic T Lymphocytes

**DOI:** 10.3389/fimmu.2022.894306

**Published:** 2022-05-03

**Authors:** Brienne McKenzie, Roxana Khazen, Salvatore Valitutti

**Affiliations:** ^1^ Institut National de la Santé et de la Recherche Médicale (INSERM) U1037, Centre de Recherche en Cancérologie de Toulouse (CRCT), Université de Toulouse III-Paul Sabatier, Toulouse, France; ^2^ Department of Pathology, Institut Universitaire du Cancer-Oncopole de Toulouse, Toulouse, France

**Keywords:** cytotoxic T lymphocytes, lytic synapse, tumor resistance, regulated cell death, immunological synapse

## Abstract

CD8^+^ cytotoxic T lymphocytes (CTLs) are the main cellular effectors of the adaptive immune response against cancer cells, which in turn have evolved sophisticated cellular defense mechanisms to withstand CTL attack. Herein we provide a critical review of the pertinent literature on early and late attack/defense events taking place at the CTL/target cell lytic synapse. We examine the earliest steps of CTL-mediated cytotoxicity (“the poison arrows”) elicited within seconds of CTL/target cell encounter, which face commensurately rapid synaptic repair mechanisms on the tumor cell side, providing the first formidable barrier to CTL attack. We examine how breach of this first defensive barrier unleashes the inextinguishable “Greek fire” in the form of granzymes whose broad cytotoxic potential is linked to activation of cell death executioners, injury of vital organelles, and destruction of intracellular homeostasis. Herein tumor cells deploy slower but no less sophisticated defensive mechanisms in the form of enhanced autophagy, increased reparative capacity, and dysregulation of cell death pathways. We discuss how the newly discovered supra-molecular attack particles (SMAPs, the “scorpion bombs”), seek to overcome the robust defensive mechanisms that confer tumor cell resistance. Finally, we discuss the implications of the aforementioned attack/defense mechanisms on the induction of regulated cell death (RCD), and how different contemporary RCD modalities (including apoptosis, pyroptosis, and ferroptosis) may have profound implications for immunotherapy. Thus, we propose that understanding and targeting multiple steps of the attack/defense process will be instrumental to enhance the efficacy of CTL anti-tumor activity and meet the outstanding challenges in clinical immunotherapy.

## Introduction

CD8^+^ cytotoxic T lymphocytes (CTLs) are highly sensitive effector cells of the adaptive immune system that identify and kill infected or transformed target cells in an antigen-specific manner. CTLs are equipped with a diverse array of biological “siege weapons” designed to penetrate exterior defenses, infiltrate target cells, and ultimately trigger tumor cell death from within through a combination of irrecoverable homeostatic perturbation and widespread intracellular proteolysis. Nonetheless, CTLs face substantial resistance from tumor cells, which have built a formidable fortress of defense mechanisms that must be overcome in succession for the attack to succeed. The dynamic interplay between CTLs and targets is the subject of this review.

## Choreography and Outcome of CTL/Target Cell Dynamic Encounters

### CTL/Target Cell Encounters

Upon encountering a potential target cell, migratory CTLs form transient conjugates mediated by the engagement of adhesion molecules such as lymphocyte function-associated antigen 1 (LFA-1) on CTLs and intercellular adhesion molecule 1 (ICAM-1) on target cells (1). During this phase, CTLs scan the target cell surface in an actin cytoskeleton-dependent manner (2) and, in the absence of antigenic recognition, rapidly disengage from their targets and re-acquire migratory behavior (3, 4). Alternatively, upon engagement of T cell receptors (TCR) by peptide-MHC class I complexes on the target cell surface, CTLs display actin cytoskeleton polymerization and LFA-1 conformational changes, leading to increased affinity for ICAM-1. As a consequence, CTLs slow down or stop their migration and establish prolonged contacts with target cells [reviewed in (2, 5, 6)].

One intriguing characteristic of the CTL response to antigenic stimulation is its dual activation threshold. While a strong antigenic stimulation is required for clonal expansion and cytokine production by CTLs (7, 8), as few as 1–10 specific peptide-MHC complexes displayed on the target cell surface suffice to trigger CTL-mediated cytotoxicity (9–11). This exquisite sensitivity enables a rapid shoot-to-kill response immediately upon detection of a target, prior to activation of the full cascade of molecular events (e.g. *de novo* synthesis of TNFα and IFNγ) associated with a sustained CTL response. Recent studies using single-molecule localization microscopy have confirmed the formation of high-density TCR-CD3 nanoclusters upon antigen recognition (even at low antigen concentrations) and this observation may underlie the CTL’s exquisite sensitivity to antigenic stimulation (12).

Antigen recognition by CTLs triggers the formation of a specialized signaling area named the immunological synapse (IS). Initially, the term IS was coined to describe the intercellular communication occurring at the contact site between CD4^+^ helper T cells and antigen presenting cells (APCs) (13–15). More recently, the term IS has been extended to include a wide range of immune cell interactions (7, 16–19). In CTLs, the dual activation threshold is reflected by the formation of two distinct synapses: the lytic synapse (LS) and the stimulatory synapse (SS). The term LS refers to molecular re-arrangements occurring during cytotoxicity (such as lytic granule polarization and docking at the CTL/target cell contact site) that are detectable in CTLs under conditions of both low and high antigenic stimulation. The term stimulatory synapse (SS) refers to the concentric large-scale segregation of surface molecules and signaling components characteristic of a mature IS and occurs only with target cells that provide the strong antigenic stimuli required for cytokine production (7). This dichotomic classification of synapses does not negate the continuous dose-dependent CTL activation process, in which several biological responses are progressively activated with increasing dose of antigen. Rather, it is an operational classification of these specialized signaling areas, underlining how synapses do not always exhibit the prototypic concentric structure based on large-scale molecular segregation, but rather their spatial configuration is a manifestation of an ongoing activation process. In line with this operational classification, additional studies put forth the notion that concentric ISs, characterized by the formation of distinct supramolecular activation clusters (SMACs) as they were originally described in helper T cells (15), are dispensable for killing activity (7, 11, 20).

**Figure 1:**
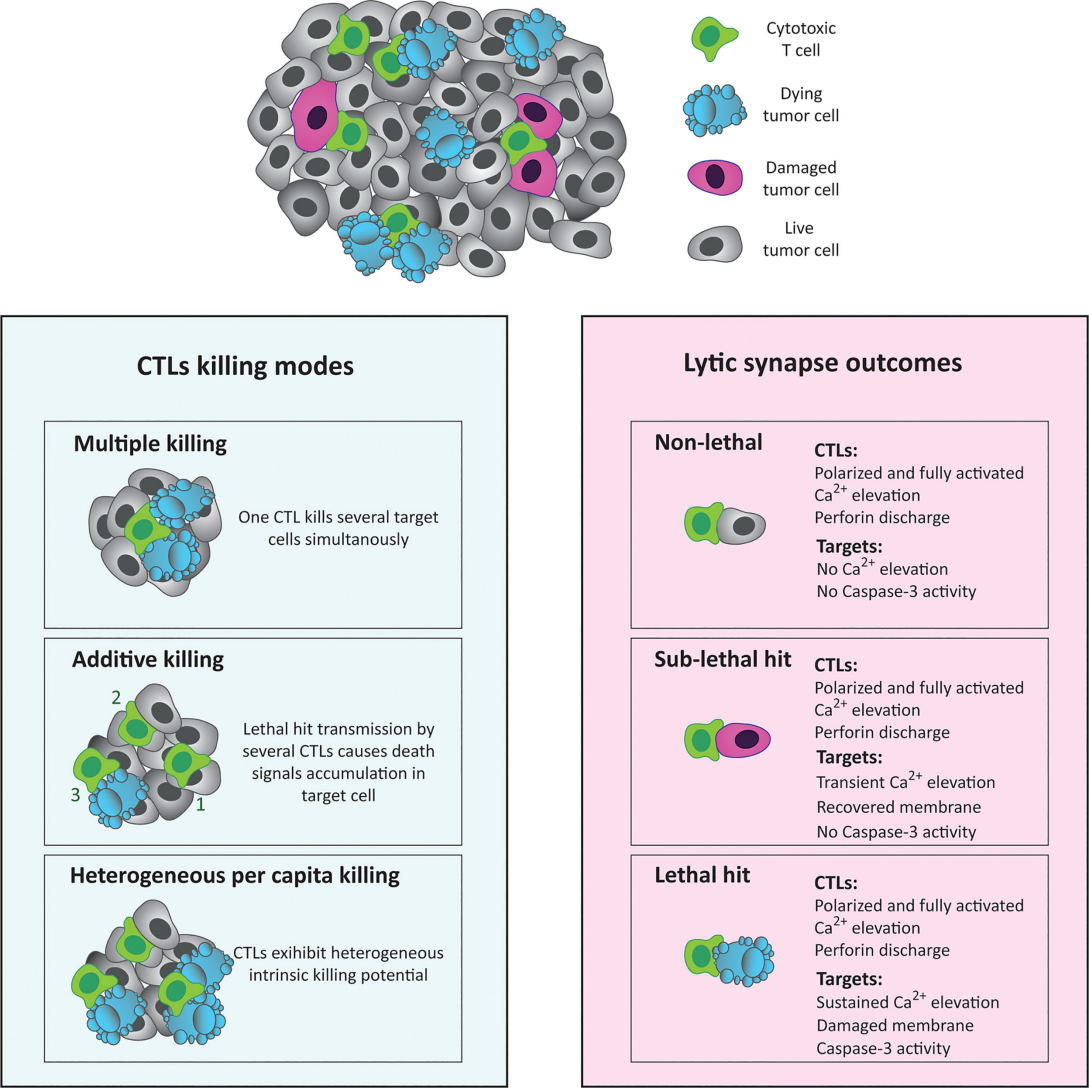
Different scenarios of CTL/target cell interaction. The left panel depicts different modes of CTL-mediated killing. CTLs eliminate tumor cells *via* a combination of killing modes, including multiple killing (one CTL kills several targets) or additive killing (several CTLs kill one target through the accumulation of intracellular damage). Furthermore, CTLs exhibit heterogeneous killing capacities ranging from high to low per-capita killing potential. The right panel illustrates individual outcomes at the lytic synapse between a given CTL and target cell. These encounters can be divided into three categories: non-lethal (in which full CTL activation does not trigger any response in target cell), sub-lethal (in which the target cell receives a CTL death signal but manages to resist the lethal outcome), and at last the lethal encounters (in which a CTL accomplishes complete annihilation of the target cell).

The ISs formed by CTLs are endowed with a high degree of plasticity and may be rapidly formed and disassembled during multiple encounters with target cells. For instance, an individual CTL can establish a stable SS with a target cell providing strong antigenic stimulation and simultaneously kill other target cells offering low antigenic stimuli by forming multiple LSs (21). This phenomenon has been defined as “multiple killing” and is at least in part responsible for the observed capacity of CTL to kill outnumbering target cells as discussed below (**Figure 1**). Sequential killing, wherein the CTL disengages from the first target cell in order to form a LS with a different target cell, can also lead to similar outcomes. For instance, chimeric antigen receptor (CAR) T-cells that co-express both a conventional TCR and a CAR have also been shown to engage in multiple killing behaviors when either the TCR or the CAR was engaged, with serial killing accounting for approximately 20% of killing events (22). Interestingly, mitochondrial translation was recently shown to be required for the sustained serial killing ability of CTLs, a phenomenon that depends upon “refueling” of CTLs with newly synthesized cytolytic proteins (23).

It is noteworthy that a functional LS requires the involvement of adhesion molecules such as LFA-1. It has been shown that productive LFA-1 engagement is essential for secretion and directed release of lytic granules (24, 25). In this respect, the dynamic physical features of the cell-cell contact sites can play an important role in the adhesiveness of the IS and the efficacy of CTL lytic function (26). In fact, following IS formation, CTLs exert mechanical forces towards their target in order to improve perforin pore formation and target cell annihilation (27–29).

LS formation provides a platform to facilitate the execution of a variety of cell-death inducing mechanisms, collectively referred to as “lethal hit delivery”. Depending upon the nature of the cell death pathway being engaged (discussed below), lethal hit delivery can be elicited within seconds after CTL/target cell encounter or evolve over a period of hours/days (30). Once the lethal hit is delivered, CTLs can detach from dying target cells, re-acquire their motility, and bind to new target cells. Jenkins et al. have provided evidence that CTL detachment from the target is a cell death-dependent process; a failure or deficiency in perforin-mediated killing can increase the dwell time before detachment from target cells, which can increase undesirable side effects such as production of excess cytokines (31). It should be noted, however, that because of the high degree of CTL motility, in particular in 3D culture conditions, target cell death is not strictly required to promote CTL detachment (30).

Early studies based on cytotoxicity measurements at low effector/target (E/T) ratios, followed by live cell imaging approaches, revealed that a single outnumbered CTL can kill multiple target cells *in vitro* (32), highlighting the impressive killing capacity of CTLs (**Figure 1**). A more recent *in vitro* study accompanied by computer-assisted modelling of CTL/target cell interaction further illustrated the capacity of outnumbered CTL to kill multiple targets (33). *In vivo* studies based on two-photon microscopy imaging of live tissues and computational analysis of CTL/target cell dynamics elegantly verified the multiple killing phenomenon, although killing appeared to occur at a slower rate *in vivo* than *in vitro* (34–36). Another recent study also confirmed that CTLs can perform serial encounters with target cells in 3D *in vitro* cultures and revealed that, under these experimental conditions, tumor cells accumulate damage during sequential encounters with different CTLs and initiate a cell death process only upon reception of several hits in close sequence (**Figure 1**) (30). A similar phenomenon of “additive killing” was observed in virus-infected fibroblasts interacting with cognate CTLs *in vivo* (34). Using intravital imaging, Khazen et al. highlighted the functional heterogeneity of CTLs inside the tumor microenvironment, illustrating that while a subset of CTLs were able to perform simultaneous killing of different target cells, others established sub-lethal contacts with multiple target cells encountered sequentially (37). The process of multiple killing therefore has two main endpoints. On one hand, it allows CTLs to kill many target cells that are intrinsically sensitive to cytotoxicity. On the other hand, sequential CTL/target cell encounters can overcome the resistance of refractory target cells.

A further key feature of CTL-mediated cytotoxicity is the considerable heterogeneity of the “per capita killing” exhibited by individual CTLs (**Figure 1**). Live cell imaging of individual human CTLs belonging to clonal populations that had been confined in micro-chambers together with outnumbering target cells showed a per capita killing varying from 1 to 12 targets during an overnight period (8). Computer-assisted analysis of overnight killing assays performed at very low E/T ratios verified this highly variable per capita killing and revealed the intriguing phenomenon that per capita killing was significantly affected by CTL density (33).

The molecular mechanisms generating such heterogeneous killing behaviors during the sustained phases of CTL/target cell interaction are presently elusive. Nonetheless, is interesting to note that super killing capability (i.e. the capacity of an individual CTL to kill many target cells) is not necessarily inherited by the super-killer’s daughter cells; upon re-stimulation and clonal expansion, an individual super-killer cell generates a progeny of daughter cells endowed with different killing capabilities (8). This observation suggests that the heterogeneous killing behavior of individual CTLs is stochastically generated during cell division. Results showing that lytic granules are stochastically and asymmetrically distributed in nascent daughter cells during human CD8^+^ T cell mitosis, as well as the demonstration that LFA-1 is likewise unequally distributed to progeny, are in line with this hypothesis (38, 39).

As reported above, heterogeneous killing behavior has also been demonstrated in a mouse model in which cytotoxicity was investigated in the tumor microenvironment using live two-photon microscopy. In this study, many CTL/tumor target cell contacts appeared to be “null”, while others resulted in limited damage of the target cells and relatively few were fully cytotoxic (37). It is conceivable that the heterogeneous killing behaviors reported in the different studies can derive from two main components, each one predominating over the other depending on the system in which cytotoxicity was studied. On one hand, heterogeneous killing efficacy can be derived from the stochastic generation of more or less “armed” CTLs during clonal expansion. On the other hand, individual tumor cells can present a stronger or weaker resistance to the attack of one or more CTLs. The stationary stochastic generation of CTLs endowed with heterogeneous killing potential at each cell division can be instrumental in randomly generating short-lived CTL cohorts harboring functional heterogeneity which are therefore more suited to face heterogeneous target cell populations.

Another important feature of CTL functional heterogeneity is that it can be markedly influenced by the microenvironment in which CTLs encounter their targets. Using intravital imaging, Michonneau et al. reported strong cytotoxicity by CTLs located in the liver while CTLs in the lymph nodes displayed a lower killing activity (40). Such anatomical heterogeneity was also observed for CAR-T cell therapy of B cell lymphoma (41). It is becoming increasingly clear that lethal hit delivery is not a homogeneous, rapid on/off phenomenon as initially considered, but rather is a multi-step, multi-faceted and, in some cases, sustained phenomenon that differs in choreography and outcome at each CTL/target cell encounter.

### The Rapid and Late Mechanisms of CTL-Mediated Cytotoxicity

The most rapid pathway used by CTLs to kill their target cells is perforin/granzyme-mediated cytotoxicity. Very rapidly after productive TCR engagement and, as mentioned above, even in the presence of weak antigenic stimulation, CTLs secrete the pore-forming protein perforin, the potent proteases granzyme A and B, and other proteases stored in the cytoplasmic granules (named lytic granules) at the LS (42, 43). Penetration of granzymes into target cells downstream of perforin-mediated target membrane perforation triggers complex and interconnected cell death pathways, which have different impacts on the immune response as detailed in later sections (44–47).

The development of ultra-rapid high-resolution techniques for live cell imaging has made it possible to assess the time elapsed between initial CTL/target cell contact and lytic granule secretion, revealing that this process is very rapid. It was initially demonstrated that within minutes after antigen recognition: i) lytic granules converge *via* a microtubule minus end-directed movement towards the microtubule organizing center (MTOC) of the CTL; ii) the MTOC is re-polarized towards the LS. The combination of these two processes brings a large fraction of lytic granules beneath the plasma membrane where they dock and fuse following a short and rapid microtubule plus end-directed movement (48–50).

Recent studies based on 4D imaging (3D plus time) provided a tomography view of LS dynamic architecture during lethal hit delivery, allowing for the precise measurement of the time required for CTL lytic machinery repolarization (51, 52). These studies showed that, in mouse CTLs, centrosome docking at the LS is complete within 5 minutes after initial TCR-coupled [Ca^2+^]_i_ in a large fraction of CTL/target cell conjugates and that lytic granules converge towards the LS during the following minutes to be secreted within an area of reduced actin density. The cortical actin network has been proposed to act as a physical barrier limiting lytic granule access to the plasma membrane and thus its synaptic depletion favors lytic granule secretion (51, 52). Accordingly, it has been reported that actin recovery at the synapse leads to termination of lytic granule secretion by CTL (53). An impact of actin network on lytic granule secretion has been shown also in the LSs formed by NK cells (54, 55). In NK cells, a dynamic network of actin cytoskeleton characterized by stochastic displacement of filaments with formation and disappearance of cortical actin at the LS has been described. This Arp2/3 and myosin IIA-dependent actin dynamism is instrumental to allow lytic granules to percolate through dynamic actin pores to reach the plasma membrane (56).

While the process of MTOC repolarization and granule convergence towards synapse has been shown to be very rapid, monitoring Ca^2+^ entry into target cells at high time resolution as a marker of plasma membrane perforation provided the surprising result that lytic granule secretion can start even earlier than MTOC re-positioning at the LS. Pore formation-dependent Ca^2+^ entry into target cells was indeed detected as early as 30-40 seconds after human CTL contact with target cells in many conjugates (57, 58), while other studies on human NK cells have shown perforation of the target cell membrane (as detected by propidium iodide penetration) within a similar time frame (59). These results are intriguing as they imply that the entire process of lethal hit delivery comprising TCR-coupled signal transduction, Ca^2+^-dependent lytic granule secretion, and perforin-mediated pore formation can occur within seconds, making CTL-mediated cytotoxicity an extraordinarily rapid biological phenomenon. These findings are compatible with precise measurements of signal transduction initiation following TCR engagement based on photoactivation of cognate pMHC complexes in mouse antigen presenting cell/CD4^+^ T cell conjugates. This approach showed a substantial progression through the TCR signaling cascade in less than 10 seconds after photoactivation, making it conceivable that a few lytic granules might be secreted by CTL within a few seconds (60).

A corollary of these findings is that the secretion of at least some lytic granules by each individual CTL can be uncoupled from MTOC re-polarization and centrosome docking at the LS, thus conferring extraordinary flexibility to lytic granule secretion and allowing a CTL to kill multiple target cells encountered simultaneously (21). The observation that centriole deletion in CTLs, while altering microtubule architecture, has surprisingly no effect on lytic granule polarization and directional secretion is in line with these observations and supports the notion that a non-centrosome-dependent lytic granule secretion pathway exists in CTLs (61).

The molecular mechanisms by which some lytic granules are secreted in the absence of MTOC re-polarization are presently elusive. It is conceivable that microtubules (MT)-initiation sites (62) might be formed at the IS during the first seconds following productive TCR engagement, leading to microtubule nucleation at the synaptic area and docking of few nearby lytic granules. As will be discussed in the following sections, while ultra-rapid lytic granule secretion confers flexibility and efficacy to the CTL killing behavior, this rapid exocytosis might also be detrimental for killing efficacy under some circumstances (57).

The above-described perforin-based cytotoxic events are all based on the rapid formation of LS at the contact site between CTL and target cells and the release of soluble perforin and granzymes into the synaptic cleft within seconds after cell-cell contact. In addition to this thoroughly investigated mechanism of lethal hit delivery, recent findings revealed that cytotoxicity might also occur *via* a delayed mechanism based on the release of molecular aggregates of lytic components and additional bioactive molecules enrobed by a glycoprotein shell. These supramolecular aggregates have been named SMAPs (Supra Molecular Attack Particles) (63, 64). SMAPs are released during the 60-90 minutes following TCR productive engagement and serve as autonomous killing entities as they remain structurally compact and biologically active after their release and binding to the extra-cellular matrix. The SMAPs, which have been identified in both CTL and NK cells (63–65), operate during an intermediate time period between the extremely rapid synaptic perforin/granzyme cytotoxicity and the death receptor-mediated cytotoxicity that can continue for hours and days after TCR triggering. The efficacy of SMAPs against cytotoxicity-resistant targets and their potential as pharmacological anti-tumoral agents are currently under intense investigation. It is interesting to note that beyond their lytic potential, released SMAPs might also play additional roles in amplifying or shaping the immune response. The observation that these entities contain chemokines suggests that they might also serve as chemotactic bio-diffusers relevant for recruiting additional effector cells to the site of CTL antigen recognition. The capacity of killer cells to release particulate supramolecular aggregates is not unique in the immune system. For instance, mast cells are also known to exteriorize their granule content on the plasma membrane and to release particulate supramolecular structures upon degranulation (66).

As mentioned earlier, in addition to the perforin/granzyme pathway, CTLs exert their cytotoxic activity through cell surface tumor necrosis factor (TNF) receptor family members including Fas ligand (FasL) and tumor necrosis factor-related apoptosis-inducing ligand (TRAIL) molecules. These are either expressed on the surface of CTLs or released as exosome membrane-bound death ligands (67, 68). Upon IS formation, FasL or TRAIL bind to their cognate receptors (Fas and TRAIL-receptor, respectively) present on the surface of the target cell. This engagement induces cleavage of pro-caspase 8 and 10 in target cells, activating the extrinsic apoptotic pathway as discussed below (69–71). Several studies suggest that slower kinetics characterize death receptor-mediated killing and referred to this as a slow killing mechanism (72). In fact, under resting conditions, few FasL molecules are expressed on the surface of CTLs, and at least 15 minutes post-TCR stimulation are required for FasL to be significantly upregulated on CTLs’ surface; continuous stimulation of T cells induces a *de novo* synthesis of this protein that peaks after 2-4 hours (73). The coexistence of a rapid low-threshold release of stored FasL with a slower FasL synthesis pathway requiring several hours suggests that CTLs combine different waves of rapid and slow FasL expression to better overcome target cell resistance (74).

The exact relevance of death receptor pathways in eliminating tumors is still under investigation. How CTLs utilize and regulate fast and slow cytotoxic mechanisms is also currently unclear. Hassin et al. provided evidence that these two pathways work in concert to mediate successful CTL cytotoxicity. In particular, FasL could restore the lytic action of late-stage poor perforin−expressing CTL (72). In addition, Prager et al. showed that during the serial encounter of target cells, NK cells switch from perforin/GzmB to death receptor-mediated killing (75).

All in all, although a clear picture of CTL-mediated cytotoxicity has not been drawn, available data strongly suggests that CTLs deploy both cellular and cell-free killing weapons at different time points upon encountering target cells. Such cooperative activity among different lytic components can be instrumental for the accomplishment of complete and durable tumor eradication.

## Choreography of the Target Cell Response to CTL Attack

### Intracellular Consequences of CTL Attack

The deployment of cytotoxic molecules from CTLs is finely orchestrated, and the target cell response to attack is equally nuanced, involving a high degree of spatiotemporal coordination and multiple waves of defense mechanisms with different kinetics. In order to appreciate the defense mechanisms at play during tumor cell response to CTL attack, it is first necessary to define the molecular effects of CTL-derived cytotoxic molecules.

Inside the target cell, one can identify two main mechanisms of CTL attack, each of which must overcome different and formidable defensive barriers. First is the engagement of intracellular regulated cell death (RCD) pathways by CTL-derived cytotoxic molecules, which directly drives RCD. Second is the catastrophic disruption of intracellular homeostasis beyond the target’s reparative capacity, which indirectly drives RCD. Together, these complementary strategies form a framework within which to conceptualize the diverse mechanisms of CTL attack.

### Direct Engagement of RCD Pathways by CTL-Derived Cytotoxic Molecules

RCD involves the engagement of specific molecular machinery within the target cell to execute an intentional cell death program, typically in response to excessive intracellular or extracellular perturbations (76). RCD is distinguished from accidental cell death (ACD) on the basis that ACD is instantaneous, catastrophic, and cannot be delayed or prevented by pharmacological or genetic means (76). Twelve RCD modalities have been identified (comprehensively reviewed elsewhere (76), each characterized by specific molecular and morphological characteristics. To date, four modalities have been implicated in target cell death upon CTL attack: intrinsic apoptosis (77), extrinsic apoptosis (73, 78), pyroptosis (79–81) ferroptosis (82). These are outlined in **Table 1**.

**Table 1:** Molecular and morphological features of different regulated cell death modalities in the context of CTL attack.

	Intrinsic Apoptosis	Extrinsic Apoptosis	Pyroptosis	Ferroptosis
**Triggering event**	• Disruption of intracellular homeostasis • Direct cleavage of caspases by granzymes	• Ligation of death receptors (e.g. Fas, TRAIL-R1/2) by cognate ligands (FasL, TRAIL)	• Proteolytic cleavage and activation of gasdermins	• Disturbance in metabolic pathways that limit formation of toxic lipid ROS
**Initiator molecules**	• Caspase-9 or granzymes	• Caspase-8 or -10	• Upstream proteases (granzymes or caspases)	• Redox-active free iron (Fe^2+^) or iron-containing lipoxygenase enzymes that oxidize membrane phospholipids
**Executioner molecules**	• Caspase-3 and -7	• Caspase-3 and -7	• Gasdermin family of pore-forming proteins	• Toxic lipid ROS derived from membrane phospholipids containing oxidized polyunsaturated fatty acid chains
**Death mechanism**	• Widespread intracellular proteolysis • Systematic demolition of cellular components	• Widespread intracellular proteolysis • Systematic demolition of cellular components	• Fatal membrane rupture following gasdermin pore formation	• Membrane phospholipids are oxidized by redox-active iron (Fe^2+^) to form toxic lipid ROS • Toxic lipid ROS fatally disrupt the plasma membrane
**Membrane features**	• Intact plasma membrane • PS exposure • Formation, maturation, and budding of apoptotic bodies	• Intact plasma membrane • PS exposure • Formation, maturation, and budding of apoptotic bodies	• Loss of membrane integrity following formation of gasdermin pores • Formation of membrane blebs/pyroptotic bodies • Rupture of pyroptotic bodies	• Accumulation of toxic lipid ROS at the plasma membrane • Loss of membrane integrity
**Molecular features**	• Mitochondrial permeabilization • Cytochrome C release • TUNEL positivity • DNA laddering • Cleavage of caspase-3/7 substrates	• TUNEL positivity • DNA laddering • Cleavage of caspase-3/7 substrates	• Release of intracellular components and inflammatory mediators (e.g. damage-associated molecular patterns, cytokines, etc.) upon membrane rupture	• Iron-dependent membrane oxidative damage and loss of lipid peroxide repair mechanisms (e.g. GPX4) • Mitochondrial abnormalities (shrinkage and loss of mitochondrial cristae)
**Additional notes**	• Secondary necrosis is possible *in vitro *(loss of plasma membrane integrity)	• Secondary necrosis is possible *in vitro *(loss of plasma membrane integrity)	• Other features that resemble apoptosis including DNA damage, TUNEL positivity, PS exposure, ROS production and mitochondrial damage have been noted in some systems	• Can be inhibited by iron chelators and lipophilic antioxidants
**Inflammatory?**	• No (except secondary necrosis)	• No (except secondary necrosis)	• Yes	• Yes

The classical mediators of apoptosis are the caspase family of cysteine-aspartic proteases, which systematically dismantle the cell through regulated intracellular proteolysis. *Intrinsic apoptosis* is driven by irrecoverable perturbations to intracellular homeostasis, which disrupt the balance of pro-apoptotic (e.g. Bax/Bak) and anti-apoptotic (e.g. Bcl-2) regulatory proteins, leading to mitochondria permeabilization, cytochrome C release, and activation of caspase-9, which in turn activates caspases-3 and -7 (76). Although the induction phase of intrinsic apoptosis is highly asynchronous across a population of cells [ranging from minutes to days following exposure to apoptotic stimulus (83)], high resolution single-cell imaging has demonstrated that the cytochrome C release phase is tightly confined to a 5 minute window (84). Within this time, cytochrome C release propagates throughout the cell in a spatially coordinated wave, initiated from a single or multiple distinct mitochondrial clusters (85). Crucially, apoptosis may be reversible at this stage (83), which offers opportunities for apoptosis resistance mechanisms to be engaged. *Extrinsic apoptosis* by contrast is initiated by ligation of plasma membrane death receptors (e.g. Fas/CD95) by their cognate ligands, which triggers the assembly of an intracellular death-receptor complex that facilitates activation of caspase-8/10 upstream of caspase-3/-7. Both intrinsic and extrinsic apoptosis converge upon activation of executioner caspases-3/7, which cleave an array of intracellular substrates, leading to precisely choreographed cellular demolition and emergence of key phenotypic characteristics such as DNA fragmentation, phosphatidylserine (PS) externalization and membrane blebbing. This final executioner phase has a restricted duration, averaging 96 minutes (83) and cannot be rescued following removal of apoptotic stimuli (83). Classically, apoptotic cells retain plasma membrane integrity throughout the demolition process until they are cleared by phagocytes, but *in vitro* end-stage apoptotic cells eventually rupture through a process called secondary necrosis. Previously assumed to be a passive process, recent studies have demonstrated that secondary necrosis is an active process facilitated by gasdermin E (86), a pore-forming executioner protein best known for its role in pyroptosis (described below), which may render apoptotic cell death inflammatory *in vitro*.

Granzyme B directly engages with RCD pathways through cleavage and activation of initiator and executioner caspases upon CTL attack; this may occur either through direct proteolytic cleavage or indirectly through the cleavage and activation of upstream caspases (87–89). Cleavage of caspase-3 by granzyme B generates a p20 fragment that requires a second cleavage event generating the p17 fragment to achieve full activity (90). This second cleavage event is constitutively blocked by the inhibitor of apoptosis (IAP) family proteins, until inhibition is released through the Bid-Smac/Diablo pathway; thus granzyme B-mediated caspase-3 cleavage requires cooperation with host apoptotic machinery and is vulnerable to fail when such machinery is inactivated (89–92). Upstream of the caspases, granzyme B can also alter the crucial balance of pro- and anti-apoptotic regulatory proteins; for instance, GzmB can directly activate pro-apoptotic Bcl-2 family members such as Bid in < 2 min (93), causing mitochondrial depolarization and release of cytochrome C (89, 93–97). The direct engagement of cell death machinery is one reason whereby a protease like granzyme B with a relatively restricted number of substrates can drive cell death so rapidly and robustly (77). It is worth noting, however, that the granzyme B substrate profile is not identical between species (89) and may be concentration-dependent (77), highlighting the need to validate findings in the human context and at physiologically relevant concentrations.

Although most early studies supported the notion that CTL-induced target cell death was apoptotic in nature, it is important to consider that alternative RCD modalities were not well-defined until recently, and that the apoptosis assays employed were not particularly specific (79). The term “apoptosis” was broadly used to distinguish RCD from ACD (at the time simply called “necrosis”) on the basis of criteria such as blebbing morphology and caspase-3 activation. Although this was a useful distinction, the historic application of the term “apoptosis” to target cell death upon CTL attack does not imply that it meets the stringent molecular criteria for apoptosis as it is defined today, nor that other related RCD modalities have been excluded. Many classical morphological and molecular features of apoptosis such as membrane blebbing, caspase-3/6/8/9 activation, PARP cleavage, PS exposure, mitochondrial permeabilization, and DNA fragmentation can be shared with other RCD modalities, and thus conventional apoptosis assays such as AnnexinV and TUNEL staining are not specific for apoptosis (98–104). That is not to say, however, that apoptosis is not an important mechanism of cell death upon CTL attack; in all likelihood, the mechanism of target cell death may be context-dependent, and subject to change based upon the characteristics of both the CTL and target cell populations.

One of the most notable non-apoptotic forms of RCD which can be engaged directly by CTL-derived lytic molecules is *pyroptosis*, a form of highly inflammatory RCD driven by gasdermin proteins (105). Gasdermins are expressed at baseline in healthy cells in an inactive conformation, wherein the C-terminal represses the pore-forming activity of the N-terminal; when gasdermin proteins are proteolytically cleaved and activated (e.g. by granzymes or by upstream caspases), the pore-forming N-terminal is liberated and translocates to the inner leaflet of the plasma membrane (106, 107). Here, gasdermin proteins assemble into multimeric pores that permeabilize the membrane, leading to cell swelling, membrane blebbing and ultimately catastrophic rupture of the plasma membrane (106, 107). Although relatively recently discovered, pyroptotic cell death has ancient origins: bacteria have been shown to express homologues of gasdermins that become lethal pore-forming toxins when released from constitutive inhibition by caspase-like proteases (108). Nonetheless, in humans, gasdermins are not universally expressed in either healthy or tumor tissue, and the presence or absence of these key executioner proteins remains a crucial determinant of a target cell’s ability to undergo pyroptosis (109).

As it pertains to cancer therapy, pyroptosis has been shown to be instrumental in promoting therapeutically beneficial anti-tumor immunity in the context of both chemotherapy and immunotherapy; however, excess pyroptosis can be associated with inflammatory side-effects (80, 81, 110, 111). Many cell-death inducing agents (including chemotherapeutics and cytokines) that were previously assumed to function through the induction of apoptosis have now been shown to actually activate pyroptosis in cells which express functional gasdermins (110–112) and crucially, many side effects of cancer therapy are observed in cell types and tissues that are particularly prone to pyroptosis (111, 113). Elucidating the cell death mechanism of different chemotherapeutic and immunotherapeutic approaches remains a pressing clinical need, and such an understanding will undoubtedly lead to greater clarity in predicting the efficacy and side-effects of different clinical approaches.

Multiple members of the gasdermin family can be activated by CTL-derived proteases (either directly or through upstream caspases) and compelling evidence has begun to accumulate for the role of pyroptosis in CTL attack (79–81). Specialized atomic force microscopy has revealed pores on the plasma membrane of patient-derived leukemic B cells after attack by CD19-recognizing CAR T cells (80). GSDME was subsequently identified as the pore-forming toxin, and its activation was shown to be granzyme-B-dependent; CAR T cell therapy was shown to induce GSDME-mediated pyroptosis *in vivo* (80). Other studies have also provided powerful evidence for GSDME-mediated pyroptosis in the clearance of tumors by CTLs and have demonstrated that granzyme B can directly cleave GSDME to release its active N-terminal domain, in addition to activating GSDME indirectly through caspase-3-mediated cleavage (81). CTL-derived granzyme A has been shown to cleave and activate GSDMB, which mediates highly inflammatory pyroptotic death in target cells (79). Inducing expression of GSDMB in target cells substantially increases susceptibility to granzyme A-mediated target cell pyroptosis *in vitro* and *in vivo* (79). The identification of non-apoptotic RCD modalities, which share important similarities with apoptosis but are driven by different molecular executioners, provides a natural explanation for “caspase-independent apoptosis” and other atypical patterns of target cell death observed anecdotally over the last several decades.

IFNγ represents another mechanism by which CTLs can directly modulate host cell death machinery. IFNγ has been shown to upregulate expression of cell death receptors (e.g. *Fas* and *TNFR1*) and pro-apoptotic mitochondrial regulators (e.g. *Bak*) within 1-4 hours, which sensitizes target cells to both intrinsic and extrinsic apoptosis (114). IFNγ also down-regulates genes involved in inhibition of apoptosis (e.g. *Bcl2* and *galectin3*) as well as those involved in survival and cell cycling (e.g. *CDK2*) (115), skewing the intracellular signaling environment towards an anti-proliferative pro-apoptotic phenotype. CTL-derived cytokines including IFNγ and TNFα can prime target cells for pyroptosis through increased expression of gasdermin family members (79), and IFNγ priming substantially increases the vulnerability of cells to pyroptosis through the granzyme A- GSDMB pathway. Importantly, recent genome-wide CRISPR assays verified IFNγ -responsive genes as key components of the CTL resistance gene signature (116, 117), verifying the role of IFNγ as a central mediator of CTL toxicity. TNFα can also directly trigger GSDMC-mediated pyroptosis through the activation of caspase-8; PD-L1 in this circumstance has been shown to translocate to the nucleus and drive expression of GSDMC, which is cleaved by caspase-8 (112). Although this mechanism was not studied in the context of CTLs specifically, this new mechanisms of TNFα-induced cytotoxicity may prove relevant in the context of sustained CTL attack.

### Irreversible Disruption of Cellular Functions and Homeostasis by CTL-Derived Cytotoxic Molecules

In addition to engaging cell death pathways directly, CTL attack also initiates a program of multi-organelle damage aimed at irreparably destroying core cellular functions and homeostasis. Mild deviations to intracellular homeostasis elicit a cellular stress response designed to re-establish homeostasis, while large deviations are injurious to the cell and may directly engage inflammatory and/or cell death pathways. The disruption of key cellular functions upon CTL attack, combined with the failure of defense mechanisms responsible for re-establishing homeostasis, are key elements of the successful CTL attack.

The program of granzyme-mediated damage to organelles has been characterized as a “post-caspase apoptotic pathway” (118) since it is not dependent upon activation of either initiator or executioner caspases. However, many granzyme-mediated cleavage events are not inherently lethal, and it requires substantial accumulated toxicity to overcome the reparative capacity of the target cell. While granzyme B is the only CTL-derived lytic molecule with direct proteolytic activity against caspases, other granzymes can participate in intracellular proteolysis events aimed at disrupting intracellular functions.

Substantial evidence has accumulated for damage to the nucleus following CTL attack, which cannot be attributed solely to caspase-3/7. Following perforation events, target cells display reduced nuclear envelop integrity, illustrated by leakage of nuclear-localized proteins into the cytoplasm after CTL contact (30), a process thought to be mediated by the caspase-independent cleavage of nuclear lamina proteins by granzyme A and B (119), as well as granzyme B-mediated cleavage of nuclear matrix proteins such as NuMA (120). CTL attack also disrupts nucleosome organization and condensation of chromatin through cleavage of histone proteins by granzymes; both DNA replication and repair are also inhibited through the inactivation of PARP1 (an early DNA damage sensor), Ku70 (involved in non-homologous end joining) and topoisomerase-1 (resolves DNA over-winding) by multiple granzymes (121–123). CTL attack can also initiate DNA fragmentation through proteolytic cleavage of ICAD/DFF45 by granzyme B and M, which releases the constitutively repressed endonuclease DFF40 (124, 125). Granzyme A can also activate the endonuclease NM23-H1 indirectly through cleavage and inactivation of its inhibitor (the SET complex); activated NM23-H1 generates single-stranded nicks in DNA, which is then further degraded by the SET complex-associated exonuclease Trex1 (126, 127). Clearly, CTL-derived lytic molecules have the capacity to inflict substantial damage upon the host nucleus; downstream activation of caspase-3/7 during CTL attack can also contribute to nuclear damage (128), and the two pathways likely converge to promote irrecoverable DNA destruction. The extent to which such damage is lethal depends not just on the extent of damage inflicted, but also upon the capacity of the tumor cell to recognize irrecoverable damage and initiate an appropriate RCD response.

CTL attack can also effectively disrupt cytoskeletal organization. For example, granzyme B mediates the cleavage of Rock II and α-tubulin (129, 130), which may affect the target cell ability to coordinate its defensive response.

CTL-derived granzymes also drive mitochondrial damage, ROS production, electron transport chain (ETC) interference, and disruption of mitochondrial membrane potential, through various mechanisms (71, 77). Granzyme A has been shown to directly induce mitochondrial damage and lead to ROS production through the cleavage of ETC complex I proteins, interfering with NADH oxidation and resulting in the production of superoxide anions (131, 132). Granzymes have been shown to penetrate the mitochondria in a Sam50-, Tim22-, and HSP70-dependent fashion, which facilitates their disruption of the ETC and resultant production of ROS (133).

IFNγ has demonstrated pro-apoptotic effects through induction of ROS and nitric oxide, though tumor cells are not universally susceptible to IFNγ-mediated cell death (134, 135). Interestingly, a recent study has highlighted the specific role of ferroptosis following IFNγ-induced oxidative perturbation upon CTL attack. *Ferroptosis* is a recently identified RCD modality characterized by lethal lipid peroxidation; the cell death process is caspase-independent, iron-dependent, and involves extensive lipid peroxidation leading to a fatal accumulation of toxic lipid peroxides and “biological rusting” of lipid membranes (76, 136, 137). Specific executioner proteins analogous to the proteases involved in apoptosis or the pore-forming proteins found in pyroptosis have not been identified; however, the main endogenous inhibitor of ferroptosis is glutathione peroxidase 4 (GPX4), which limits lipid peroxidation by reducing lipid hydroperoxides to harmless lipid alcohols (76). In the context of CTL attack, IFNγ was shown to sensitize tumor cells to ferroptosis by down-regulating the expression of SLC3A2 and SLC7A11, key regulators of cysteine homeostasis whose inhibition in turn leads to disrupted cysteine uptake and lipid peroxidation (82). A more recent study has provided important mechanistic insight into this process, implicating a cooperation between CTL-derived IFNγ and arachidonic acid in the induction of ferroptosis through the Acyl-CoA synthetase long-chain family member 4 (ACSL4) pathway (138). This reveals that CTLs can dramatically reprogram lipid metabolism in target cells through IFNγ, exploiting the accumulation of toxic lipid metabolites and the failure of lipid peroxide repair mechanisms to promote highly inflammatory target cell death (138).

The relative contribution of soluble lytic molecules versus SMAPs to intracellular damage upon CTL attack is currently unknown; interestingly, some proteases (such as caspase-1) display different substrate profiles at different concentrations (139) and thus it is conceivable that the substrate profile of granzymes might be changed when tightly complexed in a SMAP configuration. Likewise, the recently characterized multi-core granules may have different lytic molecule compositions than single-core granules, favoring specific types of intracellular damage (64). Further research will be required to understand the extent to which cellular localization and proteolytic activity of granzymes in SMAPs are different than the soluble monomers.

## Cellular Defense Mechanisms Against CTL Attack

Given the breadth of injurious effects that cytotoxic molecules have within target cells, it is not surprising that tumors develop commensurate multi-pronged defense mechanisms to counter various arms of CTL attack and mimic the rhythm of CTL killing. Studies quantifying the proportion of lethal CTL/tumor cell encounters both *in vitro* and *in vivo* have collectively revealed that relatively few CTL/tumor cell interactions are lethal, even in the context of successful antigen presentation, CTL degranulation, and target cell perforation/calcium flux (**Figure 1**) (30, 37, 41). The ability of CTLs to successfully eradicate tumors may become even further reduced over time, as constant immune editing systematically removes more susceptible immunogenic cells and drives the clonal expansion of more resistant populations, restricting intratumor genomic diversity (140).

**Figure 2:**
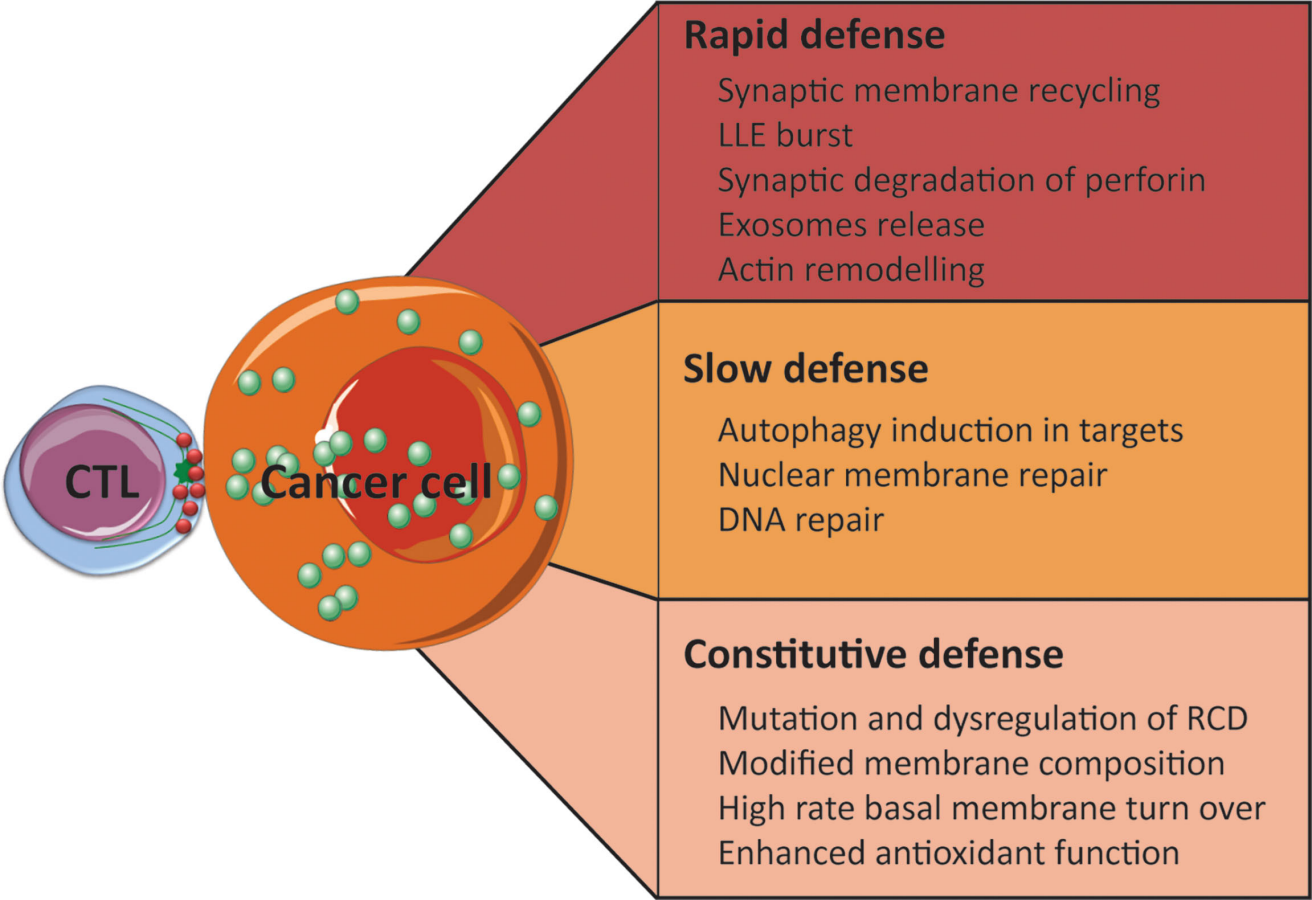
Tumor cells develop various escape mechanisms to survive CTL attack. These mechanisms can be divided into three categories: rapid, slow, and constitutive. Several examples of each category are outlined above.

Broadly speaking, resistance mechanisms can be divided into two main categories. First are the inducible defense mechanisms, which are engaged specifically upon attack by an individual CTL (e.g. membrane repair upon perforation), and these can be subdivided into rapid and slow mechanisms. Secondly are the constitutive defensive properties (e.g. inactivating mutations in cell death proteins), which may be acquired or strengthened gradually at the population level as a result of immune editing over time, but which are assumed to be pre-existing upon the attack of an individual CTL. These are summarized in **Figure 2**.

### Inducible Defense Mechanisms

#### Ultra-Rapid Defense Mechanisms

Ultra-rapid defense mechanisms are designed to neutralize cytotoxic molecules in the IS and to trigger the immediate engagement of membrane repair pathways in order to remove perforin pores from the membrane and limit the influx of granzymes. It has been shown that upon full activation of CTLs, perforin accumulates more readily on the membrane of sensitive target cells than melanoma cells, which is associated with decreased accumulation of granzyme B inside the tumor cell (141). Tumor-derived lysosomal cathepsins released into the IS can degrade soluble perforin (141), providing one mechanism for limiting the influx of cytotoxic molecules. In this way, tumor cells mimic some of the strategies adopted by CTLs to protect themselves from their own cytotoxic molecules (142), though it is worth noting that the role of cathepsins in protecting CTLs from bystander toxicity is not universally accepted (143). One mechanism for removing perforin pores once they have formed is the ultra-rapid Ca^2+^-dependent synaptic lysosomal/late endosomal (LLE) membrane repair pathway, which is engaged extremely rapidly (within seconds) upon CTL attack (44, 57, 141). Upon perforation, melanoma cell lysosomes are relocated towards the IS, and this exposure of lysosomes on the melanoma cell surface serves to remove the damaged membrane and reduce CTL-mediated cytotoxicity in a SNAP-23-depenedent manner (141). Deacidification of the lysosomal compartment effectively disables this defense mechanism (141). Importantly, this process of synaptic membrane repair is ultra-rapid and calcium-dependent; high spatio-temporal resolution single-cell imaging has demonstrated that a calcium signal propagates outwards from hotspots in the IS within milliseconds and that calcium chelation drastically increases CTL-mediated cytotoxicity by inhibiting synaptic repair mechanisms (57). In a similar scenario, actin remodeling has also been shown to mediate breast cancer cell resistance to NK cell-derived cytotoxic molecules (144, 145); in these studies, a live F-actin probe was utilized to demonstrate the massive accumulation of actin at the IS in resistant but not susceptible target cells and this synaptic actin accumulation occurred very rapidly (<2min) (144). Interestingly, the actin response persisted throughout the entire contact time between the NK cell and the tumor cell and dissipated following the detachment of the attack NK cell (144).

Release of exosomes from melanoma cells also constitutes a rapid tumor cell response to CTL attack; exosomes contain an array of different molecules that may modulate the activity of CTLs including PD-L1, which increases in exosomes upon exposure to IFNγ (146). Similar results have been obtained for colorectal cancers, wherein tumor-derived microvesicles were shown to be cytotoxic to CTLs through the FasL and TRAIL pathways (147). Given that tumor cells are able to polarize their actin and lysosomal exposure responses to the IS, it seems likely that the release of exosomes could also be directional, though this has not been definitely illustrated experimentally.

#### Slower Defense Mechanisms

In addition to rapid synaptic defense mechanisms, slower defense mechanisms are engaged within minutes to hours in order to attempt to re-establish homeostasis, remove damaged organelles, and promote recovery from sublethal CTL attack. For example, induction of autophagy (which degrades damaged organelles) has been shown to favor tumor cell survival upon CTL attack (148) and these results have been strongly supported by recent genome-wide CRISPR screens *in vitro* and targeted CRISPR screens *in vivo* (116). This study identified a core set of 182 target genes (out of 123,000 guide RNAs tested) that mediate melanoma cell resistance to CTL attack, amongst which autophagy genes were particularly enriched; it was subsequently confirmed that inhibition of autophagy either genetically or pharmacologically sensitized tumor cells to CTL attack (116). However, other studies have postulated that autophagy is required for the efficacy of CTL-mediated attack and that autophagy deficiency reduces tumor cell killing (149), highlighting the yet-unresolved complexity of autophagy in CTL attack.

It has also been shown using live-cell microscopy that nuclear integrity can be restored [potentially by membrane repair complexes such as ESCRT III (150)] in minutes to hours following leakage of nuclear-localized reporters into the cytoplasm in damaged cells, within a median time of 49 minutes post-contact (30). Furthermore, engagement of DNA repair complexes (as measured by 53BPI foci formation) occurs in a substantial number of CTL:target contacts, which can persist for several hours but ultimately resolve once repair is complete (30). These observations highlight how conserved cell-intrinsic repair mechanisms play a key role in defense against CTL attack and provide a mechanism for why sequential or simultaneous interactions with multiple CTLs increases the probability of overwhelming cellular repair mechanisms. Using mathematical modeling based on live-cell imaging, it has been estimated that the “damage half-life” is on average 56.7 minutes *in vitro*; further hits to an injured target within the repair window increase the likelihood of target cell defenses being overcome and CTL attack triggering target RCD (30).

### Constitutive Defense Mechanisms

A fundamental limitation to the efficacy of CTL attack is that its arsenal converges upon target cell RCD. While granzymes can mimic certain aspects of executioner caspases, the CTL is dependent upon target cell machinery to sense and integrate the cell death signals, and then to ultimately execute the cell death process. This presents a formidable challenge in tumors since cancer cells are notoriously effective at hamstringing their own RCD machinery (151). For instance, in a comprehensive transcriptional study of 675 human cancer cell lines, pathway-based mutation aggregation demonstrated that the p53 pathway (a tumor suppressor upstream of intrinsic apoptosis that responds to intracellular stressors) was the most universally dysregulated pathway across cancer types (152) and these results were recapitulated in a genomic profiling cohort containing over 18,000 adult cancers (153). As p53 constitutes the major pathway for triggering apoptosis downstream of DNA damage, constitutive inactivation of this pathway curtails the ability of granzyme-mediated DNA damage to drive RCD.

Altered expression of both pro- and anti-apoptotic *Bcl2* family members is also well-documented (154), and dysregulation of the microRNAs responsible for regulating these proteins has been demonstrated across multiple cancer types (154). Interestingly, a novel role for Bcl-2 has also recently been identified in the regulation of pyroptosis, wherein GSDMD-bound Bcl-2 prevents the GSDMD-activating cleavage event (155). Such observations highlight how multiple RCD modalities may be blocked simultaneously by the tumor and highlight opportunities for combination therapies (e.g. with Bcl-2 inhibitors) to remove the brakes on target cell RCD following CTL attack. Proof of principle for this has been demonstrated (156), illustrating that overcoming constitutive barriers to cell death is promising in the context of immunotherapy.

Tumors can also upregulate inhibitors of apoptosis proteins (IAPs) such as XIAP, IAP1 and IAP2, which serve to inhibit caspases through either direct means (e.g. blocking the substrate binding pocket of active caspases) or indirect means (e.g. targeting active caspases for proteosomal degradation) (76). XIAP for instance has been shown to be over-expressed in most cancer cell lines (157). Catalytically inactive homologues of caspases (e.g. FLIP family proteins) can also form heterodimers with initiator caspases, blocking their autoproteolytic activation (76). Upstream of this, death receptors such as Fas have been shown to be aberrantly expressed in multiple malignancies through mechanisms such as downregulation, internalization, or mutation (often in the cytoplasmic domain that facilitates death receptor complex assembly), thus conferring resistance to FasL, a prominent weapon in the CTL aresenal (158). Non-signaling decoy receptors (e.g. the FasL-mimicking decoy receptors DcR 1-3) and soluble decoy proteins (such as osteoprotegerin) are over-expressed in many tumor types and can further impede death-receptor signaling (159). Importantly, the Fas/FasL pathway requires functional host caspases, and inactivation of these apoptotic proteins effectively neutralizes FasL-mediated killing (160).

Of course, tumors also inhibit expression of both initiator and executioner caspases directly to prevent CTL-derived cytotoxic molecules from engaging the cell death machinery; caspase-8 and caspase-3 are within the top ten most mutated RCD proteins in cancer (157). Importantly, altered executioner caspase functionality may not only impact a cell’s ability to undergo apoptosis but also pyroptosis, since GSDME-mediated pyroptosis can be driven by active caspase-3 (110, 111).

By contrast, granule-mediated killing can circumvent the requirement for host caspases in some circumstances (128, 160), illustrating the importance of redundancy in CTL killing mechanisms as a way of circumventing RCD dysregulation in cancer. Unlike in Fas/FasL-mediated apoptosis, wherein all molecular features of apoptosis are caspase-dependent, mitochondrial depolarization, membrane blebbing, and cell lysis may still be observed during granule-mediated killing in the absence of one or more functional executioner caspases (94, 118, 128). Given that pyroptosis shares several of these molecular features with apoptosis, it is conceivable that cleavage of gasdermins by granzymes, which has been recently confirmed (79, 81), may be responsible at least in part for the progression of granule-mediated target RCD in the absence of functional caspases, potentially in cooperation with other granzyme substrates.

It is worth noting however that gasdermin family members are also inconsistently expressed and subject to silencing and mutation within tumors. For example, GSDMB is infrequently expressed in cancer cell lines, except those derived from gastric cancers (79). Furthermore, GSDMB expression in primary tumors is only partially correlated to its expression in healthy tissue; further profiling of tissue samples from 75 gastric and 80 esophageal cancers revealed that only 45% of gastric tumor samples and 55% were positive for GSDMB, despite virtually all of the corresponding healthy tissue sections showing robust GSDMB expression (79). As a further barrier to pyroptosis, GSDME is also repressed in the context of cancer; it has been shown to be detectable in only ~10% of human cancer cell lines (5 of 60 lines tested in the NCI-60 panel) (111). Although expressed in many healthy tissues, GSDME can be effectively silenced in the context of cancer by promoter methylation, and expression can be restored through a methyltransferase inhibitor (161). Mutation of gasdermin proteins is also observed in the context of tumorigenesis. For instance, interrogation of the TCGA database demonstrated that GSDME had a high prevalence of mutations, which were especially concentrated around the caspase-3 cleavage site; 20 of 22 cancer-associated GSDME mutations tested were shown to inhibit its function (81). Clearly, such repression and mutational burdens present a formidable barrier to the success of CTL-driven immunotherapies that rely upon pyroptosis in target cells; however, they also provide opportunities to increase susceptibility to killing through upregulation of gasdermins through strategies such as inhibition of promoter methylation (e.g. methyl transferase) or IFNγ pre-treatment. Unfortunately, however, the IFNγ pathway itself may be subject to dysregulation in cancer, thus curtailing CTL efficacy. A recent genome-scale CRISPR-Cas9 screen looking for targets whose inhibition increases sensitivity to CTL killing demonstrated that mutations in the IFNγ pathway confer a significant survival advantage to target cells (162). Other groups have shown that defects in IFNγ signaling confer resistance to anti-CTLA4 therapy (163).

Several studies have validated that perforin binding and pore formation is impaired on the surface of transformed cells (141, 164), and multiple mechanisms may contribute to this phenomenon. In addition to the degradation of perforin on the membrane by lysosomal cathepsins (141), which is an ultra-rapid resistance mechanism, constitutive properties of cancer cells may impair perforin pore formation. For instance, melanoma cells have been shown to have constitutively high membrane turnover (141), a mechanism that may provide dual protection not only against perforation from external pore-forming toxins (such as perforin) but also potentially against internal pore-forming executioner proteins (such as gasdermins). One must also consider how the altered plasma membrane properties of cancer cells may impair perforin binding and render tumor cells particularly refractory to perforation during CTL attack (164). On the other hand, it has been proposed that certain biophysical properties of cancer cells might enhance their susceptibility to CTL-mediated attack. It has been reported that myocardin-related transcription factors (MRTFs) overexpression rigidifies actin filaments, which renders targets more susceptible to CTL cytotoxicity (165).

Transformed cells, particularly migrating or metastasizing ones, are prone to membrane damage as a result of trafficking through the dense ECM, and as such they compensate through the enhanced expression of membrane repair proteins (such as Annexin2) that orchestrate membrane fusion and wound healing (166). Upon membrane injury, annexins facilitate the accumulation of actin at the wound perimeter, which is a crucial step in wound closure that has also been implicated in defense against CTL attack (166). It has further been demonstrated that the plasma membranes of cancer cells have unique phospholipid compositions that include a particular enrichment of externalized PS on the outer leaflet, which is enhanced under conditions of oxidative stress (167, 168); interestingly, exposure of PS on the surface of CTLs has been shown to trap perforin in a dysfunctional, non-pore-forming conformation and it has been speculated that the enrichment of PS on the tumor cell membrane may provide enhanced protection against perforin (169). Additionally, perforin is less capable of penetrating lipid bilayers that are rich in sphingomyelin and cholesterol (167, 169). Although not universally observed, an increase in plasma membrane cholesterol has been shown in a variety of cancers (167, 168). Given the sensitivity of perforin to target membrane composition (169), it is conceivable that a membrane composition that is suboptimal for perforin binding and pore formation provides an additional barrier to successful perforation by CTLs. Whether transformation-induced alterations to the plasma membrane lipid composition likewise make tumor cells more refractory to their own pore-forming RCD proteins such as gasdermins (which are also sensitive to lipid composition) remains to be determined.

As an additional protective mechanism, cancer cells are equipped to withstand a greater degree of disruption to intracellular homeostasis than can other cells, not only because the RCD mechanisms that would typically be engaged upon loss of homeostasis are constitutively disabled but also because pathways to support survival in suboptimal conditions are constitutively engaged (170). For instance, tumor cells express high levels of proteins with antioxidant functionality to help them withstand ROS damage (171). The master regulator of the antioxidant response, the transcription factor nuclear factor erythroid 2-related factor 2 (Nrf2) and the antioxidant enzymes under its control (such as glutathione S-transferases and UDP-glucuronosyltransferases) can be constitutively activated in tumors through interactions with oncogenes such as *KRAS* and *MYC* (171). *NRF2* is also mutated in a variety of cancers, leading to constitutive stabilization of the transcription factor in the nucleus (172). Such adaptations severely undermine the ability of CTL-generated ROS to exert lethal effects upon tumor cells.

The autophagy network is also crucial for integrating stress signals, recycling damaged organelles, and driving cell death in the event that intracellular stress exceeds the reparative capacity of the autophagic network; however this network is highly perturbed in tumorigenesis through mutation and dysregulation of autophagy genes, which promote cell survival under suboptimal circumstances (170, 173). Constitutively elevated levels of autophagy are observed in many cancers, and it has been shown that following exposure to otherwise-lethal stress, cancer cells can utilize their enhanced autophagic capabilities to shrink into a state of reversible dormancy, rather than dying in response to extreme stress (151, 174). Autophagy is also crucial for the removal of damaged organelles, such as ROS-producing mitochondria, and thus enhanced autophagic capacity of some tumor cells confers a formidable survival advantage (170). The ability to withstand extreme intracellular stress without dying presents a formidable obstacle to the successful eradication of target cells by CTLs. Furthermore, activation of autophagy in tumor cells has been shown to protect against lytic granule attack through multiple mechanisms *in vitro* and *in vivo*, including the direct autophagic degradation of NK -derived granzyme B in the lysosomal compartment, ultimately impairing target cell lysis (148, 175). When intracellular signaling pathways are constitutively skewed towards survival (even at the expense of genetic stability and intracellular homeostasis), a CTL faces formidable resistance even in the context of successful antigen presentation and degranulation.

Beyond this, tumor cells express constitutively high endogenous levels of serine protease inhibitors (SERPINS) such as serine protease inhibitor 9, (PI-9) which inhibits proteolytic activity of granzyme B and is associated with poor outcome and response to immunotherapy in melanoma (176–179); importantly, expression of PI-9 has been shown to increase in tumor cells in response to IFNγ, increasing the challenge posed during CTL attack (180). Recent CRISPR-Cas9 screens have validated targets such as *Serpinb9* as mediators of CTL resistance (117).

The challenge of both slow and constitutive defense mechanisms is that these mechanisms are often the same ones that provide enhanced resistance to conventional therapies such as chemotherapy and radiation, and in fact the kinetics of repair following CTL attack closely agree with recovery times following other types of physical or chemical damage (30). This indicates that although CTLs have many ways of promoting target cell RCD, they face many of the same formidable barriers as conventional therapies. While this may be perceived as a limitation, it is also an opportunity for combination therapy to additively overcome cell defense mechanisms using classical therapies along with immunotherapy approaches.

Moreover, while chemotherapy and radiotherapy tend to activate a limited range of RCD pathways, CTLs are capable of circumventing blockades of any individual cell death pathway; a target cell that is highly resistant to apoptosis, for example, may still be effectively killed by one of the five different pyroptosis pathways that may be engaged in sequence or in parallel during CTL attack. For instance, it has been shown that caspase-3-deficient cancer cells are still vulnerable to CTL-mediated RCD through alternative mechanisms, though certain elements of the cell death phenotype (e.g. DNA fragmentation) are lost (94).

The characterization of these defense mechanisms is of immense clinical importance, due to the significant population of patient non-responders to cancer immunotherapy. Above and beyond this, there is an accumulating body of literature to suggest that failed apoptosis, and more specifically failed CTL or NK cell attack, can actually benefit the cancer cells, promoting migration, metastasis, acquisition of stem cell-like features, and increased tumor aggressiveness (181, 182). Failure to kill target cells specifically can lead to prolonged hypersecretion of proinflammatory cytokines by CTLs that fail to detach from a resistant target, increasing the probability of inflammatory side-effects (31). As such, the ability to identify resistance mechanisms to immunotherapy and prevent failed CTL attack is a pressing clinical need. While successful checkpoint inhibitor strategies have brought immense optimism to the field of immunotherapy by “releasing the brakes” on CTLs, even a fully activated CTL still faces immense challenges in initiating cell death in an environment biased towards tumor cell survival.

## Concluding Remarks

Going forward, it will be important to bear in mind several principles regarding heterogeneous CTL attack modalities and target cell resistance to CTL. Firstly, given the plethora of cellular defense mechanisms faced by CTLs attacking tumor cells, it is likely that a tailored immunopharmacological approach may be required clinically to sensitize target cells to CTL attack; alternatively, non-cellular delivery approaches that circumvent CTL-specific defense mechanisms (e.g. SMAPs) and might be equipped “ à la carte” with different cytotoxic weapons are worth investigating. Although CTLs are equipped with a truly impressive array of diverse weaponry, tumor cells retain sophisticated defense mechanisms for evading attack on both the ultra-rapid, slow, and constitutive time scales, such that even in the context of effective antigen presentation and target recognition, the CTL attack may be blunted.

Secondly, the evidence in the literature for non-apoptotic target cell death following CTL attack is accumulating to the point where it is difficult to justify the continued use of “apoptosis” indiscriminately as a synonym for target RCD. Recent research in oncology has provided unprecedented insight into the profound implications of pyroptosis in cancer development and treatment [extensively reviewed elsewhere (183)]. It is likely that cell death modality is heterogeneous, dependent upon the properties of both the CTL and tumor cell populations; it has been suggested that depending upon the diversity of cell death executioner molecules expressed, different target cells may respond very differently to attack by identical CTLs (79), a phenomenon which only increases in complexity when we also consider the heterogeneity on the CTL side of the synapse.

It is important to consider that target cell death may simply resist categorization into a single cell death modality. Given the plethora of different pathways activated during CTL attack, it is likely that target cell death combines elements of different modalities, an observation that has already been noted in other studies wherein RCD is chemically induced (98, 100, 104, 184). In a physiological system such as CTL attack wherein the attack mechanism is multimodal, this is even more likely to be true. As such, it may be informative to remove the preconception that target cell death should adhere to the prescribed set of morphological and molecular characteristics that define a given cell death modality, and instead embrace the full complexity of the intracellular response to the attack of heterogeneous cohorts of CTLs. Such a perspective would comfortably accommodate earlier observations in the field of target cell death that combined both apoptotic and non-apoptotic features.

Lastly, it is important to acknowledge that each cell death modality is associated with its own regulatory and resistance mechanisms, and as such broadening our understanding of target cell death mechanism during CTL attack may help to uncover previously underappreciated resistance mechanisms and therapeutic targets. In the current era of immunotherapy, there is an urgent need on the one hand to potentiate cell-mediated and cell-free mechanisms of cytotoxicity, and on the other hand, to understand the mechanisms of resistance ranging from synaptic defense to cell death resistance in order to address unmet clinical needs.

## Author Contributions

BM, RK, and SV wrote and edited the manuscript. RK designed the figures. All authors contributed to the article and approved the submitted version.

## Funding

This work was supported by grants from the European Research Council (ERC) under the European Union’s Horizon 2020 research and innovation program (Grant agreement No. Syn- 951329) and Bristol-Myers Squibb (No CA184-575), and from INSERM institutional funding. The funders had no role in preparation of the manuscript.

## Conflict of Interest

The authors declare that the research was conducted in the absence of any commercial or financial relationships that could be construed as a potential conflict of interest.

## Publisher’s Note

All claims expressed in this article are solely those of the authors and do not necessarily represent those of their affiliated organizations, or those of the publisher, the editors and the reviewers. Any product that may be evaluated in this article, or claim that may be made by its manufacturer, is not guaranteed or endorsed by the publisher.
